# High-Performance Non-Volatile InGaZnO Based Flash Memory Device Embedded with a Monolayer Au Nanoparticles

**DOI:** 10.3390/nano11051101

**Published:** 2021-04-24

**Authors:** Muhammad Naqi, Nayoung Kwon, Sung Hyeon Jung, Pavan Pujar, Hae Won Cho, Yong In Cho, Hyung Koun Cho, Byungkwon Lim, Sunkook Kim

**Affiliations:** Department of Advanced Materials Science and Engineering, Sungkyunkwan University, Suwon 16419, Korea; alinaqi@skku.edu (M.N.); nybabono@skku.edu (N.K.); wjdtjdgus2@skku.edu (S.H.J.); p.b.pujar@gmail.com (P.P.); winther99@naver.com (H.W.C.); jjo304@naver.com (Y.I.C.); chohk@skku.edu (H.K.C.)

**Keywords:** non-volatile memory device, flash memory device, three-terminal memory device, IGZO, monolayer Au nanoparticles

## Abstract

Non-volatile memory (NVM) devices based on three-terminal thin-film transistors (TFTs) have gained extensive interest in memory applications due to their high retained characteristics, good scalability, and high charge storage capacity. Herein, we report a low-temperature (<100 °C) processed top-gate TFT-type NVM device using indium gallium zinc oxide (IGZO) semiconductor with monolayer gold nanoparticles (AuNPs) as a floating gate layer to obtain reliable memory operations. The proposed NVM device exhibits a high memory window (ΔV_th_) of 13.7 V when it sweeps from −20 V to +20 V back and forth. Additionally, the material characteristics of the monolayer AuNPs (floating gate layer) and IGZO film (semiconductor layer) are confirmed using transmission electronic microscopy (TEM), atomic force microscopy (AFM), and x-ray photoelectron spectroscopy (XPS) techniques. The memory operations in terms of endurance and retention are obtained, revealing highly stable endurance properties of the device up to 100 P/E cycles by applying pulses (±20 V, duration of 100 ms) and reliable retention time up to 10^4^ s. The proposed NVM device, owing to the properties of large memory window, stable endurance, and high retention time, enables an excellent approach in futuristic non-volatile memory technology.

## 1. Introduction

Previously, non-volatile memory (NVM) devices based on thin-film transistors (TFTs) gained great potential because of minimum power dissipation to retrain the stored data and their conformal compatibility with complementary integrated circuits and electronic chips [1,2,3,4,5,6]. For typical NVM devices, the programming/erasing properties depend on the applied voltage to the control gate electrode (V_program_/V_erase_) that leads to validate the memory window with the corresponding variation in the current level of a device as ‘on’ state and ‘off’ state, describing programmable and erasable constitutes of memories [7,8,9]. In this regard, floating gate NVM devices based on various semiconductors, such as two-dimensional (2D) materials (molybdenum disulfide (MoS_2_) and tungsten disulfide (WS_2_)), [3,8,10,11,12,13] oxide materials (indium gallium zinc oxide (IGZO) and zinc oxide (ZnO)) [9,14,15,16,17,18,19,20] and organic materials (pentacene, poly(3-hexylthiophene) (P3HT), and dinaphthothienothiophene (DNTT)) [21,22,23,24,25,26] have recently been studied, exhibiting high-performance memory operations with greater endurance and retention properties but are still limited at obtaining reliable reproducibility, larger memory window with on-off ratio, low-temperature processing, and easy fabrication methods.

Meanwhile, the floating gate layer is also anticipated to be a promising constituent in the three-terminal NVM device’s structure for higher charge storage capacity and suitable endurance/retention memory operations [8,9,19,20,26]. Additionally, the charge-trap layer such as HfO_2_, SiN, and redox state molecule has been introduced in the NVM device’s structure to store and erase the charge according to the memory state controlled by a swing in the threshold voltage (V_th_) [27,28,29,30,31]. On the contrary, NVM devices based on metallic nanoparticles as a floating gate have gained more advantages, such as higher tapping probability of charges, ensuring the stable probability of retention property in case of defects in the tunneling or control oxide layer, and larger memory window [8,32,33,34]. In this context, metal nanoparticles as floating gate layer in NVM devices offer a noteworthy consideration in the memory operations subjected to attain high programming or erasing properties, a greater probability of the charge retention, and preferable density of state (DOS) near the fermi-level [32,33,35,36,37,38]. Additionally, metal oxide semiconductor field-effect transistor (FET)-related NVM devices are also investigated for memory operations such as read-only memory, endurance, and retention [39,40]. However, such NVM memory devices with a floating gate, charge-trap, or MOSFET structures exhibit high memory performance but still, appropriate fabrication methods, low-temperature processing, and high charge-storage capacity seem to be inevitable, which requires favorable processing techniques to obtain stable and reliable memory performance.

Herein, we report a low-temperature-process (≤100 °C) method to a fabricate TFT type flash memory device based on indium gallium zinc oxide semiconductor, utilizing Au nanoparticles as a floating gate layer, revealing the memory window with high charge-storage capacity and stable endurance/retention memory operations, and favorable processing techniques. IGZO semiconductor as an active material is deposited via the radio frequency (RF) sputtering technique, exhibiting high electrical performance with stable retention time. Furthermore, the status of oxygen and metal vacancies in the IGZO channel material is analyzed using x-ray photoelectron spectroscopy (XPS) with a depth-profiling function to confirm the oxidizing power effects on metals and oxygen peaks. The charge storage layer of monolayer Au nanoparticles (AuNPs) is transferred onto a tunneling layer of the proposed NVM device, assisted with a floating film of AuNPs on the water surface. The memory measurements of the presented NVM devices exhibit electrically programmable-erasable properties over a voltage range of −20 V~+20 V with a large memory window (ΔV_th_) of 13.7 V. Furthermore, the memory endurance and retention characteristics are investigated, revealing high stability and reliability with nearly 100 cycles of Programming/Erasing cycles and >10^4^ retention time, respectively, thus, enabling an advancement into NVM devices with excellent memory performances for futuristic storage electronic devices.

## 2. Materials and Methods

### 2.1. Fabrication of the Proposed NVM Device

To fabricate the proposed NVM device, firstly, the IGZO with a thickness of 60 nm is deposited on a rigid glass by radiofrequency (RF) technique and then patterned by simple lithography process in which photoresist (PR, AZGXR-601, MERCK, Kenilworth, NJ, USA) is spin-coated onto the pre-deposited IGZO film at 3000 rpm for 20 s. Then, the exposure of UV light is applied for 5 s in the presence of a specified mask and developed in the developer (AZ-300MIF) for 20 s. After patterning the PR, the pattern of IGZO film is obtained after dipping the substrate in the diluted buffer oxide etchant (BOE) for 10 sec. After IGZO film patterning, source and drain (S/D) electrodes are patterned by using a lift-off process in which lift-off resist (LOR3B, Product # G3167070500L1GL, MicroChem, Austin, TX, USA) is spin-coated at 2000 rpm for 45 s, along with the PR coating and then developed in the developer for 30 s. After patterning the LOR/PR film, the titanium/gold (Ti/Au ~20/100 nm) was deposited by using an e-beam evaporator, and the unwanted area of Ti/Au was removed by dipping the substrate in PG-remover (mr-Rem 700, Micro-Resist Technology, Berlin, Germany) for 60 s at 80 °C. After patterning the S/D electrodes, 20 nm of Al_2_O_3_ is deposited by atomic layer deposition (ALD) method at 100 °C and followed by transferring of monolayer Au nanoparticles film onto the tunneling layer of the proposed device. Here, the AuNPs that have been capped with 6-aminohexanoic acid are floated on the water surface. A self-assembled monolayer of the gold nanoparticles was transferred onto the tunneling layer. Next, the gate dielectric layer of Al_2_O_3_ (40 nm) is deposited at 100 °C by the ALD technique, and then the via of the S/D electrodes are patterned using the above-mentioned lithography method. As a final step, the control gate electrode is patterned using the above-mentioned lift-off photolithography process.

### 2.2. Material Characterizations

To analyze the material characteristics, the transmission electron microscopy (TEM), atomic force microscopy (AFM), and X-ray photoelectron spectroscopy (XPS) are measured.

### 2.3. Memory Characterizations

The electrical memory measurements have been analyzed by using a semiconductor analyzer system (Keithley, 4200 SCS, Cleveland, OH, USA) in ambient conditions.

## 3. Results and Discussion

**Figure 1:**
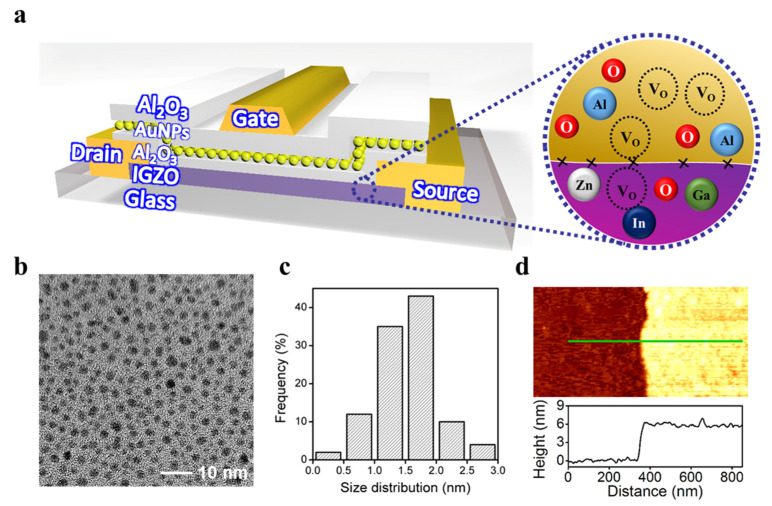
Non-volatile memory device based on the floating gate of monolayer AuNPs. (**a**) A schematic layout of the proposed floating gate NVM device. (**b**) A TEM image of monolayer AuNPs, utilized as a floating gate layer (scale: 10 nm). (**c**) A size distribution bar graph of the proposed monolayer AuNPs layer. (**d**) An AFM analysis of proposed monolayer AuNPs with a thickness distribution scale.

Particularly, NVM devices based on TFT structure have been strongly studied for optimizing excellent memory performances through various device configurations but remain challenging to acquire stable and reliable memory properties due to unfavorable processing techniques. Furthermore, the NVM device in modern technology aims to assimilate a long-life storage capability with a large memory window over a small voltage range through favorable and simple processing techniques. Our design and favorable processing technique of the proposed NVM device attempt to overcome the existing limitations of the low-temperature process by defining the top control gate structure type flash memory device, which has a great potential to obtain a large memory window and stable endurance/retention operations. The entire proposed NVM device stack based on IGZO channel material with a floating gate layer of Au nanoparticles is elaborated in Figure 1a, distinguishing the IGZO film and tunneling layer of Al_2_O_3_ as shown in the inset. Recent studies of flash memory devices show that the floating gate layer, specifically nanoparticles, is capable of impressive charge storage capacity due to its strong coupling effect with channel material, higher space for charge storing nodes, and a favorable state-of-the-art process forming nanoparticles film. Here, the AuNPs monolayer film, used as a floating gate layer, is prepared by the interfacial assembly of AuNPs [41]. Figure 1b presents a transmission electron microscopy (TEM) image, revealing a monolayer of AuNPs film without multiple stacking and spatial separation from each other. The size of the most constituent AuNPs is less than 3 nm as shown in Figure 1c. The thickness of the monolayer AuNPs, which includes a ligand shell, is measured to be 5.5 nm using atomic force microscopy (AFM), shown in Figure 1d.

**Figure 2:**
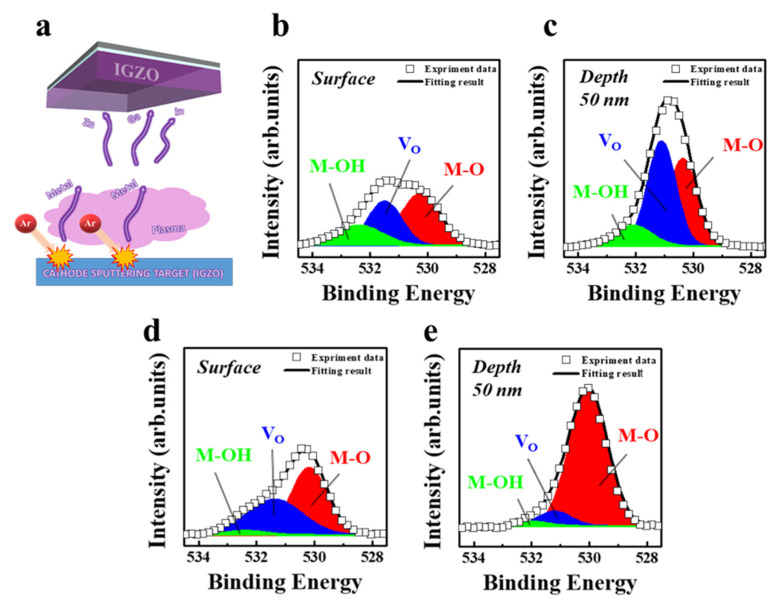
Materials analysis of the proposed IGZO semiconductor utilized in the device. (**a**) A schematic layout of the sputtering process of IGZO channel material. (**b**,**c**) An XPS analysis of IGZO film before annealing with surface and depth (50 nm) profiling method, respectively. (**d**,**e**) XPS analysis of IGZO film after annealing with surface and depth (50 nm) profiling method, respectively.

Meanwhile, the active material in the TFT-type NVM device serves to obtain high-performance memory operations with a high aspect ratio and error-free long-life retention time. Herein, the IGZO channel layer is deposited by radio-frequency sputtering (150 W, 3 mTorr) at room temperature on a rigid glass substrate. The working modes of TFTs have been artificially managed by controlling the gas ratios [Ar/O_2_~28/2 sccm] during the deposition process, as shown in the schematic layout of Figure 2a. For stably re-arranging deposited atoms and defect healing, the channel layer has been followed by thermal annealing (350 °C at 1 h) in the air atmosphere. To investigate the chemical bonding status of metal and oxygen ions before and after the thermal annealing process, x-ray photoelectron spectroscopy (XPS) technique with a depth-profiling function has been analyzed where argon ions (Ar^+^) of 200–400 eV irradiated the surface of the samples. Additionally, the chemical bonding has been characterized after the ion etching for 50 nm depth-profiling measurements with and without thermal annealing process. The oxygen spectra (O_1s_) are deconvoluted into three curves with a Gaussian fitting tool, shown in the Figure 2b,c, elaborating the XPS measurements without the thermal annealing process of as-deposited IGZO channel layer and 50 nm depth-profiling function, respectively. The results show the changes in the oxygen concentration curves in three chemical bondings distinguished by surface and 50 depth-profiling functions without thermal annealing, corresponding to the bonding of metal-oxygen (M-O) of 529.5 ± 0.3 eV, oxygen-deficient (V_O_) vacancies of 530.9 ± 0.3 eV, and metal-hydroxyl (M-OH^−^) impurity of 531.2 ± 0.3 eV, shown in Figure 2b,c. Furthermore, the change in the oxygen concentration has also been analyzed after the thermal annealing process obtained from surface and depth-profiling function using XPS analysis, depicted in Figure 2d,e, respectively. The increment in the oxygen vacancies with an unnoteworthy shift in peak has been observed after the thermal annealing process. The O_1s_ peaks from the IGZO channel layer can be generally deconvoluted into the curves from the above-mentioned three chemical bondings, corresponding to M-O peak of 529.7 ± 0.3 eV, V_O_ peak of 531.1 ± 0.3 eV, and M-OH^−^ peak of 531.4 ± 0.3 eV, as depicted in Figure 2d,e. The depth analysis of metal-oxygen binding reveals that thermal treatment induces the intensively dominant M-O bonding by effectively supplying oxygen, similarly to metal-related XPS results. These XPS results on metal and oxygen peaks confirm distinctly that the plentiful supply with high oxidizing power causes considerable effects. In the case of the IGZO channel layer, the average contribution from oxygen-deficient vacancies is significantly reduced and the formation of the unwanted M-OH^−^ bonding is suppressed.

**Figure 3:**
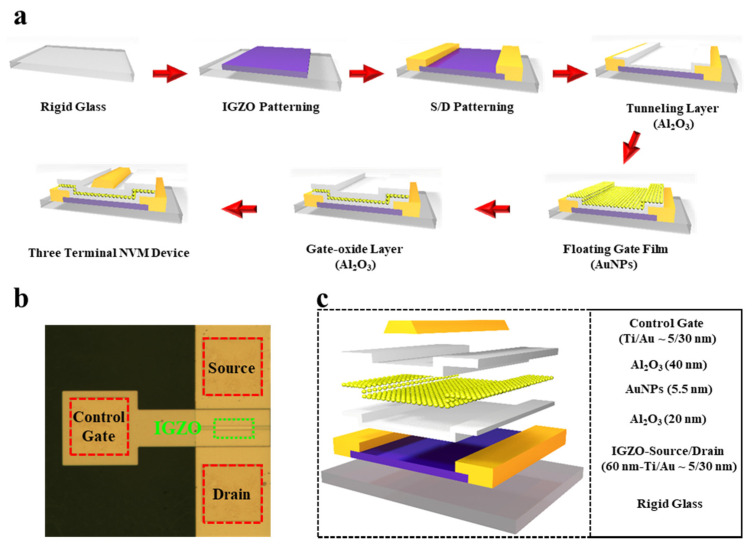
NVM device based on stacking of IGZO/Al_2_O_3_/AuNPs/Al_2_O_3_ layers. (**a**) A sequential fabrication process of the proposed NVM devices. (**b**) An optical image of the proposed NVM device. (**c**) A layer-by-layer schematic layout of the proposed NVM device along with each thickness information.

After the material characterizations of the floating gate layer and channel film, the NVM device is fabricated, and the step-by-step fabrication process is demonstrated in Figure 3a. After depositing the IGZO semiconductor film on the rigid glass, it is patterned by a simple photolithography process with specified mask design and followed by source/drain patterning using lift-off process, where titanium/gold (Ti/Au) has been utilized as metal electrodes. Next, the Al_2_O_3_ with a thickness of 20 nm as a tunneling layer of the proposed three-terminal NVM device is deposited via atomic layer deposition (ALD) process under the temperature of 100 °C and then the monolayer AuNPs film (thickness ~5.5 nm) is dispersed onto the tunneling layer of the NVM device via transferred process assisted with a floating film onto the water surface. After the clean dispersion of monolayer AuNPs, the gate dielectric layer of Al_2_O_3_ with a thickness of 40 nm is deposited, the same as the above-mentioned tunneling layer deposition. As a final step, the gate electrode is patterned using a lift-off lithography process after making the via of source/drain electrodes. Furthermore, all the steps are performed under the temperature of 100 °C and discussed in more detail in the experimental section. Figure 3b demonstrates the optical image of the proposed three-terminal NVM device with the labeling of electrodes and channel material. Additionally, the layer-by-layer stacking structure of the proposed three-terminal NVM device is described in the schematic layout of Figure 3c, revealing the thickness parameters of each layer.

**Figure 4:**
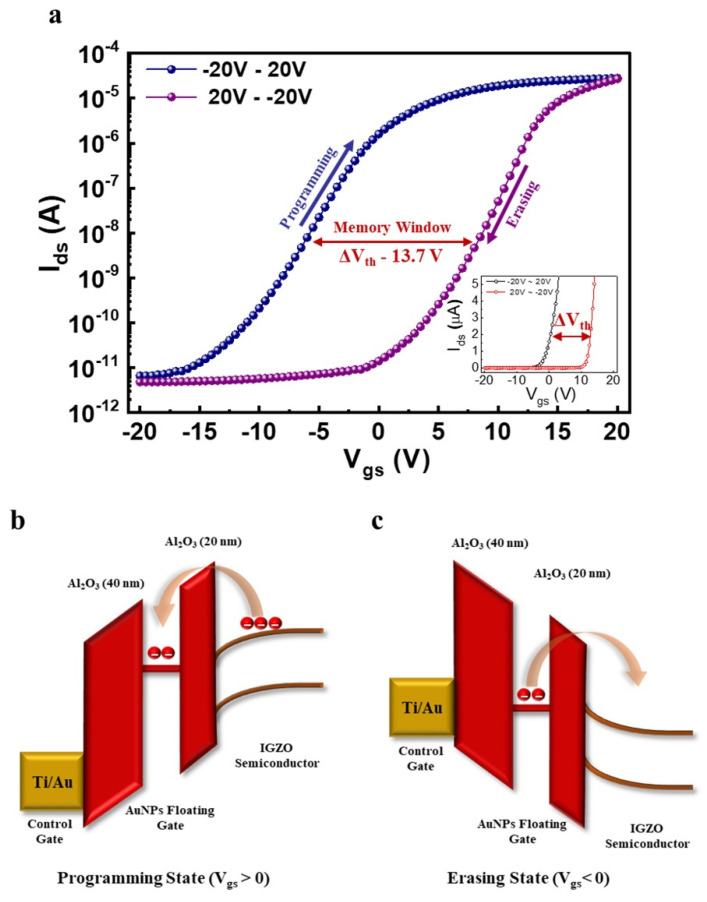
Transfer characteristics of the proposed NVM device. (**a**) A hysteresis of the transfer curve of the proposed NVM device when sweeping from −20 V to +20 V back and forth, defines a memory window of 13.7 V. (**b**,**c**) A band diagram illustration of the proposed NVM device in case of programming and erasing when V_gs_ > 0 V and V_gs_ < 0 V, respectively.

After fabrication of the proposed three-terminal NVM device, its electrical transfer characteristics are measured to observe the storage capability, where the voltage sweep is applied from −20 V to +20 V and back to −20 V at V_ds_ of 1 V as shown in Figure 4a. A large memory window (ΔV_th_) of 13.7 V is observed over a preferable range of applied voltage (−20 V to +20 V), principally initiated from a large amount of charge carriers stored in the AuNPs floating gate layer. Furthermore, the inset of Figure 4a explains the linear scale transfer curve of the proposed three-terminal NVM device, defining the clear threshold voltage difference (ΔV_th_) of programming and erasing state. The following three-terminal NVM device characteristics are extracted in case of forward bias (−20 V to +20 V), V_th_ of −4.5 V, and I_on_/I_off_ of 10^6^, revealing excellent transfer properties of the proposed IGZO-based NVM device. Furthermore, a comparison table of the proposed NVM device with the previously reported flash memory devices based on IGZO semiconductor material is demonstrated in Table 1, distinguished as following benchmark parameters such as gate stack, channel deposition method, memory window (ΔV_th_), and applied voltage range, revealing a large memory window over a small preferable voltage range (−20 V to +20 V) with easy processing methods. The proposed three-terminal NVM device memory operation is illustrated in the form of a band diagram, shown in Figure 4b,c, in case of programming and erasing states, respectively. When the control gate voltage (V_gs_) is swept from the negative voltage to the positive voltage, electrons can tunnel through the tunneling layer of Al_2_O_3_ (20 nm) as per the Fowler-Nordheim tunneling effect [7,42,43]. This way, the charge stored in the floating gate layer will shift the threshold voltage (V_th_) and result in a programming state (Figure 4b). When the control gate voltage (V_gs_) is swept from the positive voltage to the negative voltage, electrons can discharge and tunnel back to the IGZO channel, resulting in an erasing state (Figure 4c).

**Figure 5:**
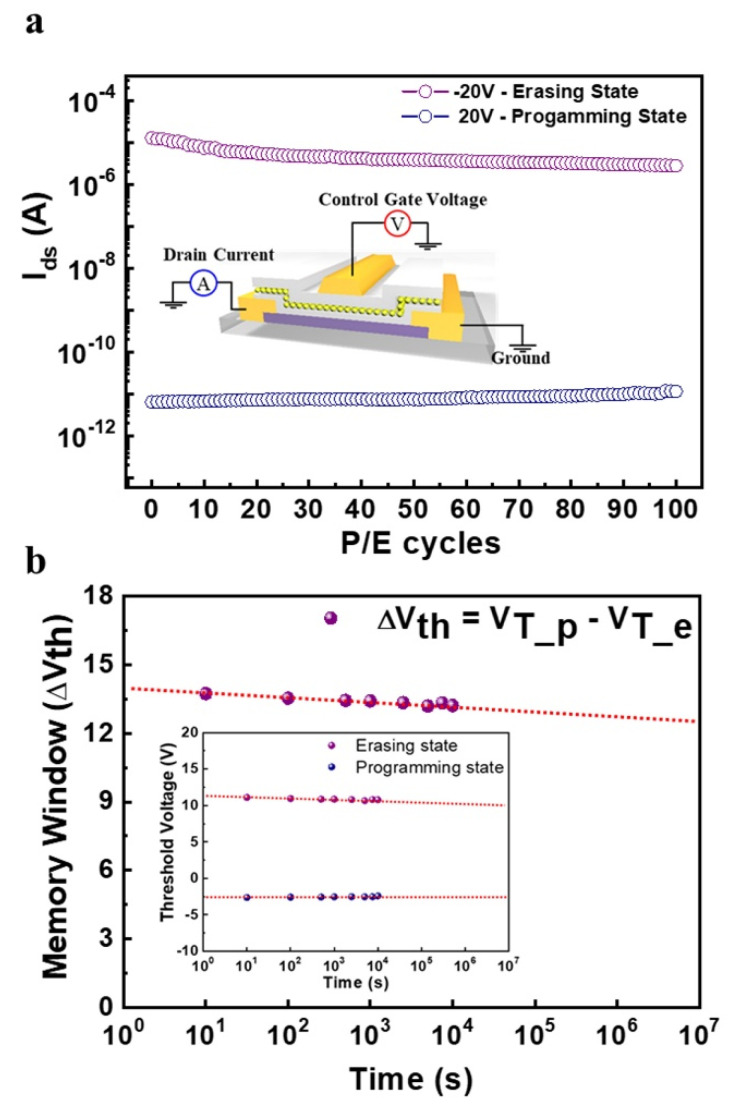
Memory operation of the proposed NVM device. (**a**) An endurance characterization of the proposed NVM device by applying a sequence of pulses (±20 V, duration of 100 ms) to the control gate at the V_ds_ of 1 V up to 100 P/E cycles. (**b**) A retention characterization of the proposed NVM device in form of a memory window (ΔV_th_) at different time scales up to 10^4^.

Next, the key figures of merit for non-volatile memory devices such as endurance and retention are evaluated to determine the stability and reliability of the device. In Figure 5a, the endurance characteristics of the proposed NVM device are analyzed by applying a sequence of pulses (±20 V, duration of 100 ms) to the control gate at the V_ds_ of 1 V, revealing stability and robustness of the device after repeated programming/erasing cycles (up to 100 P/E cycles). The endurance properties of the proposed NVM device show a well maintained high current on/off ratio of 10^5^. As a function of retained time for the proposed NVM memory device, the data retention properties are analyzed in the memory window (ΔV_th_) form as shown in Figure 5b. The threshold voltage in terms of memory window with programming/erasing states is measured at different time scales up to 10^4^ s, as shown in the inset of Figure 5b. Here, the memory window (ΔV_th_) is calculated as ΔV_th_ = V_T_p_ − V_T_e_; where V_T_p_ represents the threshold voltage at programming state and V_T_e_ represents the threshold voltage at erasing state. The extracted memory window (ΔV_th_) varies from 13.7–13.2 V after 10^4^ s retention time, estimating that a low percentage (<20%) of charges will be lost after 10 years. The results show high stability and robustness of the presented device with excellent memory operation, thus, enabling a great potential in next-generation non-volatile memory technology.

## 4. Conclusions

We have investigated the top-gate TFT-type NVM device which uses an IGZO semiconductor film and a monolayer AuNPs floating gate layer. The material characteristics of IGZO and monolayer AuNPs layers are also performed by using TEM, AFM, and XPS analysis. To fabricate the proposed NVM device, all the processes are performed at low-temperature (<100 °C) using simple and easy processing techniques. A large memory window (ΔV_th_) of 13.7 V is observed when it sweeps from −20 V to +20 V forward and +20 V to −20 V backward, depicting a high charge storage capacity of the proposed NVM device. Furthermore, programming and erasing states are explained using a band diagram followed by the Fowler-Nordheim tunneling effect. Next, memory operations such as endurance and retention are measured, revealing a stable endurance up to 100 P/E cycles and a high retention time of 10^4^. Owing to the high memory properties, the demonstrated three-terminal NVM device shows a promising approach in the field of non-volatile memory technology.

## Figures and Tables

**Table 1:** A comparison of the presented NVM device with the previously reported flash memory devices based on IGZO semiconductor material.

Gate Stacks	Channel Deposition Method	Memory Window (ΔV_th_)	Voltage Range	Reference
Al_2_O_3_/Au-NPs/Al_2_O_3_/IGZO	RF Sputtering	~13.7	−20 V–20 V	This work
Al_2_O_3_/Pt-NCs/Al_2_O_3_/IGZO	Magnetron Sputtering	~4.04	−10 V–10 V	[18]
SiO_2_/Au-NCs/SiO_2_/IGZO	RF Magnetron Sputtering	~4.7	−15 V–15 V	[44]
Ion-gel/AuNPs/IGZO	Sol-gel Process	~0.88	−9 V–9 V	[45]
SiO_2_/AuNPs/PE/HfO_2_/IGZO	RF Magnetron Sputtering	~15	−50 V–50 V	[9]
Al_2_O_3_/HfO_2_/Al_2_O_3_/IGZO	RF Sputtering	~11	−10 V–30 V	[17]

## Data Availability

The data are available on the request from corresponding authors.
